# Circulating MiR-125b as a Marker Predicting Chemoresistance in Breast Cancer

**DOI:** 10.1371/journal.pone.0034210

**Published:** 2012-04-16

**Authors:** Hongjiang Wang, Guang Tan, Lei Dong, Lei Cheng, Kejun Li, Zhongyu Wang, Haifeng Luo

**Affiliations:** Department of General Surgery, First Affiliated Hospital of Dalian Medical University, Dalian, People's Republic of China; Queen Elizabeth Hospital, Hong Kong

## Abstract

**Background:**

Chemotherapy is an important component in the treatment paradigm for breast cancers. However, the resistance of cancer cells to chemotherapeutic agents frequently results in the subsequent recurrence and metastasis. Identification of molecular markers to predict treatment outcome is therefore warranted. The aim of the present study was to evaluate whether expression of circulating microRNAs (miRNAs) can predict clinical outcome in breast cancer patients treated with adjuvant chemotherapy.

**Methodology/Principal Findings:**

Circulating miRNAs in blood serum prior to treatment were determined by quantitative Real-Time PCR in 56 breast cancer patients with invasive ductal carcinoma and pre-operative neoadjuvant chemotherapy. Proliferating cell nuclear antigen (PCNA) immunostaining and TUNEL were performed in surgical samples to determine the effects of chemotherapy on cancer cell proliferation and apoptosis, respectively. Among the miRNAs tested, only miR-125b was significantly associated with therapeutic response, exhibiting higher expression level in non-responsive patients (n = 26, 46%; *p* = 0.008). In addition, breast cancers with high miR-125b expression had higher percentage of proliferating cells and lower percentage of apoptotic cells in the corresponding surgical specimens obtained after neoadjuvant chemotherapy. Increased resistance to anticancer drug was observed *in vitro* in breast cancer cells with ectopic miR-125b expression; conversely, reducing miR-125b level sensitized breast cancer cells to chemotherapy. Moreover, we demonstrated that the E2F3 was a direct target of miR-125b in breast cancer cells.

**Conclusions/Significance:**

These data suggest that circulating miR-125b expression is associated with chemotherapeutic resistance of breast cancer. This finding has important implications in the development of targeted therapeutics for overcoming chemotherapeutic resistance in novel anti-cancer strategies.

## Introduction

Breast cancer is the leading cause of cancer and the second leading cause of cancer death in American women [1], and its incidence is increasing in many countries including China [2]. Chemotherapy is an important component in the treatment paradigm for breast cancers. However, despite a rapid shrinkage in tumor mass following chemotherapeutic cycles, the resistance of cancer cells to chemotherapeutic agents frequently results in the subsequent recurrence and metastasis of cancer [3]. To data, there is no validated sensitivity and/or resistance predictive factor available in clinical settings and the mechanisms involved in cancer cell chemoresistance are still largely unknown. Therefore, it is in urgent need to search for circulating markers and identify signaling pathways in breast cancer patients resistant to chemotherapy.

The molecular mechanisms that may contribute to chemotherapeutic resistance in breast cancers include overexpression of ATP-binding cassette transporters, anti-apoptotic factors and cell cycle deregulation [4]–[6]. However, targeting any single molecule is not sufficient to reverse chemotherapeutic resistance [7], suggesting that multiple molecular pathways may contribute to the sensitivity of breast cancer cells to chemotherapy.

MicroRNAs (miRNAs) are a group of non-coding, single-stranded RNAs of ∼22 nucleotides, which regulate gene expression by binding to the 3′-untranslated region (UTR) of target mRNAs, repressing mRNA translation or cleaving target mRNA [8]. As master regulators of gene expression, miRNAs are involved in modulating multiple cellular pathways, including cell proliferation, differentiation, and apoptosis, and thus may function as oncogenes or tumor suppressing genes [9], [10]. Importantly, circulating miRNAs have been detected as potential blood-based biomarkers for cancer detection [11]–[14]. Among them, oncogenic miRNAs, including miR-10b [15], miR-34a [16], miR-125b [17], miR-155 [18] *etc*, have been reported to be related to breast cancer. In recent years, some miRNAs have been reported to be involved in drug-resistance. Inhibition of miR-221 or miR-222 expression has been shown to sensitize MDA-MB-468 cells to tamoxifen-induced cell growth arrest and apoptosis [19]. Ectopic miR-34a expression resulted in cell cycle arrest and growth inhibition and attenuated chemoresistance to the anticancer drug, camptothecin, by inducing apoptosis [20]. To date, however, there has been no report on the role of circulating miRNAs in chemotherapeutic resistance of breast cancers.

The aim of the present study was to evaluate potential biological markers to predict outcome from adjuvant chemotherapy. Therefore, we have performed an integrative analysis of the expression of miR-10b, miR-34a, miR-125b and miR-155 in the serum of advanced breast cancer patients, with known pathological and treatment characteristics. We identified an association of circulating miR-125b level with chemotherapeutic response. In addition, the effects of overexpression and knockdown of miR-125b on chemotherapeutic resistance was investigated by *in vitro* assays.

## Results

### Circulating miRNA expression profiles and correlation with clinical outcome

**Figure 1:**
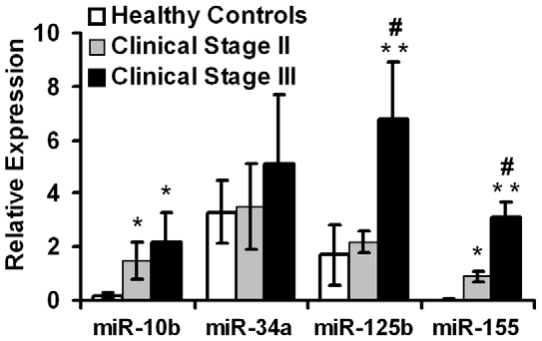
Levels of circulating miRNAs in breast cancer patients and healthy controls. Relative levels of circulating miR-10b, miR-34a, miR-125b and miR-155 were measured in healthy individuals (n = 10) and clinical stage II patients (n = 35) and stage III patients (n = 21) by quantitative RT-PCR. Data represented mean ± SD. *p<0.05, **p<0.01 compared to healthy controls; #*p*<0.05 compared to clinical stage II patients.

Expression of 4 miRNAs, miR-10b, -34a, -125b and -155, was profiled in the serum because of their established relevance to breast cancer [15]–[18]. Consistent with previous reports, the levels of miR-10b and miR-155 were significantly higher in the early stage (Stage II, n = 35) and late stage (Stage III, n = 21) breast cancer patients than in healthy controls (n = 10) (Fig. 1). In addition, the late stage breast cancer patients had significantly higher level of miR-155 than early stage patients (Fig. 1 and Table 1). Although early stage breast cancer patients had similar miR-125b level to healthy controls, late stage patients had on average 3.5-fold higher mean values of miR-125b than early stage patients and healthy controls (*p*<0.01, Fig. 1 and Table 1). Circulating miR-34 did not differ significantly in each comparison.

Table 1 depicted the correlation of the circulating miRNA levels in 56 patients, who had advanced ductal breast cancer and received pre-operative neoadjuvant chemotherapy, with clinical and histopathological factors. To determine the differences in the relative expression profiles, we performed univariate analyses of the Mann Whitney-U test. Expression levels of miR-125b and miR-155 were related to disease stage with higher miRNA levels in higher stage disease (*p*≤0.02), but lack of significant association with estrogen receptor (ER), progesterone receptor (PR) and epidermal growth factor receptor 2 (HER2) status. Furthermore, blood levels of miR-125b were significantly associated with tumor grade and lymph node metastasis of the patients (*p*≤0.04). In contrast, expression of preoperative circulating miR-10b and miR-34a did not differ significantly in each comparison.

**Table 1:** Patients' characteristics at the time of primary diagnosis of breast cancer and correlation with serum miRNA levels.

Characteristics	Subgroups	n	Mean (Standard Deviation)			
		(total 56)	miR-10b	miR-34a	miR-125b	miR-155
Clinical stage	II	35 (63%)	1.5 (0.7)	3.5 (1.6)	^**^2.2 (0.4)	*0.9 (0.2)
	III	21 (38%)	2.2 (1.1)	5.1 (2.6)	^**^6.8 (2.1)	*3.1 (0.6)
Grade	II	32 (57%)	1.7 (0.6)	3.8 (1.9)	^*^2.6 (0.7)	1.5 (0.4)
	III	24 (43%)	2.1 (1.0)	4.9 (1.2)	^*^6.1 (1.9)	2.3 (1.0)
Distant metastasis	M0	20 (36%)	1.9 (0.9)	3.9 (1.3)	3.6 (1.1)	1.7 (0.5)
	M1-2	36 (64%)	2.1 (0.9)	4.5 (2.1)	5.9 (1.8)	2.1 (1.2)
Node metastasis	N1-3	40 (71%)	1.5 (0.7)	3.2 (1.1)	*3.1 (0.5)	1.9 (1.0)
	N≥4	16 (29%)	2.3 (1.2)	5.2 (2.6)	*6.3 (1.1)	2.1 (0.9)
ER	Negative	13 (23%)	2.1 (1.1)	4.1 (1.9)	4.6 (1.3)	2.2 (1.1)
	Positive	43 (77%)	1.9 (0.8)	4.7 (1.9)	4.1 (1.1)	1.9 (0.6)
PR	Negative	19 (34%)	1.9 (0.5)	3.9 (1.5)	3.9 (0.9)	1.6 (0.5)
	Positive	37 (66%)	2.2 (1.3)	4.6 (2.1)	4.8 (1.5)	2.3 (1.2)
HER2	Negative	46 (82%)	1.7 (0.8)	3.8 (0.9)	3.8 (1.4)	2.1 (0.9)
	Positive	10 (18%)	2.0 (1.2)	4.5 (1.3)	4.2 (1.8)	1.7 (1.1)

ER, estrogen receptor; PR, progesterone receptor; HER2, human epidermal growth factor receptor 2 * *p*<0.05, ** *p*<0.01.

### Circulating miR-125b expression correlated with chemotherapeutic resistance of breast cancer patients

**Figure 2:**
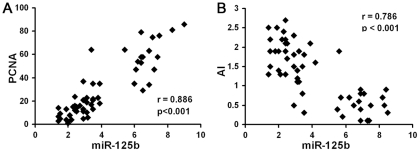
Circulating miR-125b expression correlated to chemotherapeutic response in breast cancer patients. Surgically removed tumor samples were obtained after neoadjuvant chemotherapy. (A) Percentage of PCNA positive cells was determined by immunohistochemical staining. (B) Apoptotic index (AI) was expressed as the percentage of TUNEL-positive tumor cells. Circulating miR-125b levels were determined by quantitative real-time PCR.

To determine whether the circulating miRNA expression levels were associated with chemotherapeutic efficacy, therapeutic response was evaluated by radiologic Response Evaluation Criteria in Solid Tumors (RECIST) as well as PCNA immunostaining for cell proliferation and TUNEL assay for apoptotic index in surgical specimens after chemotherapy. According to RECIST, 30 patients (54%) responded to chemotherapy with PR or CR; 26 patients (46%) were not responsive with SD or PD. Among the circulating miRNAs, only miR-125b was significantly associated with therapeutic response, exhibiting higher expression level in non-responsive patients (*p* = 0.008, Table 2). In addition, breast cancers with high miR-125b expression prior to chemotherapy had higher percentage of proliferating cells and lower percentage of apoptotic cells in the corresponding surgical specimens obtained after neoadjuvant chemotherapy (Fig. 2A and B), suggesting that miR-125b expression in breast cancers was reversely correlated with apoptosis and proliferation inhibition induced by chemotherapy. Responders and non-responders for chemotherapy exhibited similar expression levels of miR-10b, 34a and 155 (Table 2).

**Table 2:** Correlation of serum miRNA levels and response to chemotherapy.

		n	Mean (Stand Deviation)			
		(total 56)	miR-10b	miR-34a	miR-125b	miR-155
Treatment response	CR+PR	30 (54%)	1.9 (0.8)	4.1 (1.3)	^**^1.8 (0.3)	1.9 (0.5)
	SD+PD	26 (46%)	2.0 (0.9)	4.3 (1.6)	^**^7.4 (1.9)	2.1 (0.9)
PCNA	Low	35 (63%)	1.5 (0.5)	3.5 (1.2)	^**^1.9 (0.6)	1.8 (0.6)
	High	21 (38%)	2.5 (1.4)	4.7 (1.9)	^**^6.9 (2.1)	2.2 (0.9)
Apoptotic index	Low	22 (39%)	1.9 (0.7)	3.9 (1.5)	*6.3 (1.3)	2.2 (0.9)
	High	34 (61%)	2.1 (1.1)	4.5 (2.2)	*2.9 (0.9)	1.6 (1.0)

CR, complete response; PR, partial response; SD, stable disease; PD, disease progression * *p*<0.05, ** *p*<0.01

### MiR-125b expression correlated with chemotherapeutic resistance in primary breast cancer cells

**Figure 3:**
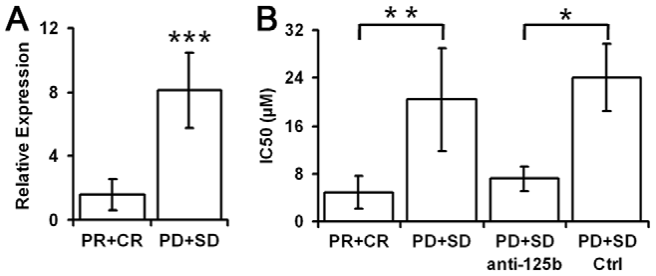
MiR-125b expression correlated with chemotherapeutic resistance in primary breast cancer cells. (A) MiR-125b expression was determined by quantitative RT-PCR. (B) Primary breast cancer cells were treated with 5-FU for 48 h with or without miRNA antisense transfection (100 nM). IC50 was calculated as concentration of 5-FU to produce 50% cell inhibition (Materials and Methods). Data represented mean ± SD of primary breast cancer cell cultures from 5 patients with partial or complete response (PR + CR) and from 4 patients with disease progression or stabilization (PD + SD), *p = 0.01, **p = 0.002, ***p<0.001.

To further determine whether expression of miR-125b might be correlated with chemotherapeutic response, we isolated primary cancer cells from the biopsies of 11 breast cancer patients obtained prior to chemotherapy and correlated miR-125b expression levels with the efficacy of chemotherapy. Among all the 11 patients who received neoadjuvant chemotherapy, 6 patients had PR or CR, while the other 5 patients had PD or SD. The miR-125b expression levels were significantly higher in breast cancers with PD/SD than those with PR/CR (8.1±2.3 fold, *p*<0.001, Fig. 3A). Similarly, the IC50 of 5-FU for breast cancer cells with PD/SD was significant higher than those with PR/CR based on the dose–response curves (4.8±2.7 vs. 20.4±8.6, *p* = 0.002, Fig. 3B). Furthermore, knockdown of miR-125b using an antisense sequence significantly decreased IC50 of 5-FU for breast cancers with PD/SD to the similar levels as those with PR/CR (Fig. 3B). These findings demonstrate that a higher expression level of miR-125b is associated with poor clinical response of primary breast cancers to chemotherapy.

### Involvement of miR-125b in chemotherapeutic resistance in breast cancer cells

**Figure 4:**
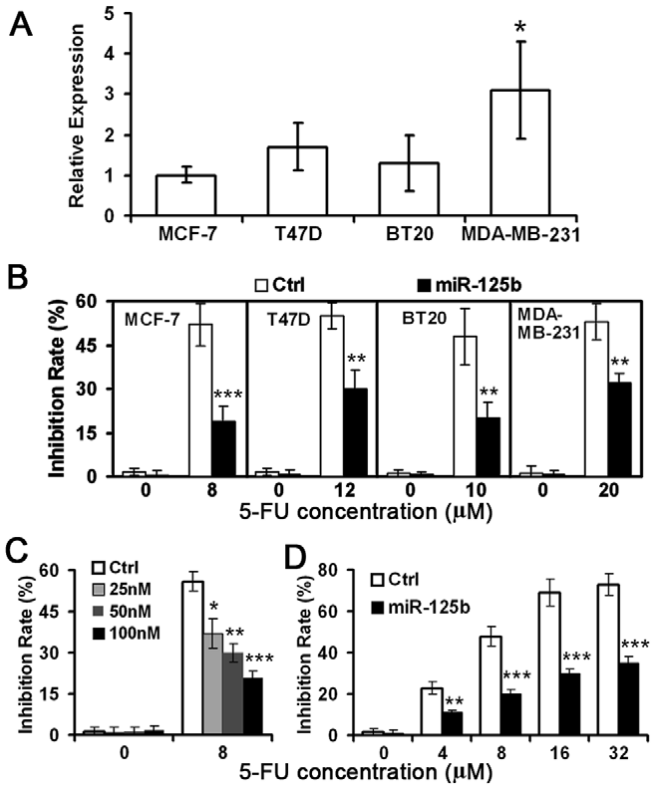
Involvement of miR-125b in chemotherapeutic resistance in breast cancer cells. (A) Relative expression levels of endogenous miR-125b were measured in breast cancer cell lines by quantitative RT-PCR. Data represented mean ± SD. *p<0.05 compared to MCF-7. (B) Breast cancer cells, MCF-7, T47D, BT20 and MDA-MB-231, were transfected with 100 nM pre-miR-125b and treated with the indicated concentrations of 5-FU for 48 h. A scramble pre-miRNA sequence served as a negative control (Ctrl). MCF-7 cells were transfected with 0, 25, 50, and 100 nM pre-miR-125b and then incubated with 8 µM 5-FU (C) or were treated with 0, 4, 8, 16, and 32 µM 5-FU for 48 h after transfection of 100 nM miRNA mimics (D). Cell variability was determined by trypan blue dye exclusion assays. Data represented mean ± SD of triplicate experiments. *p<0.05, **p<0.01, ***p<0.001 compared to Ctrl.

In order to directly evaluate the contribution of miR-125b to chemotherapeutic response, we investigated the role of miR-125b in chemotherapy resistance in breast cancer cell lines, MCF-7, T47D, BT20 and MDA-MB-231. Among these cells, MCF-7 exhibited the lowest endogenous miR-125b (Fig. 4A), whereas MDA-MB-231 cells have 3.1±1.2 (mean±SD) fold higher miR-125b expression than MCF-7 (*p* = 0.03). Based on the dose–response curves, the IC50 of 5-FU was calculated as 8 µM for MCF-7, 12 µM for T47D, 10 µM for BT20 and 20 µM for MDA-MB-231, respectively. These concentrations were used in the following experiments. MiR-125b overexpression was induced using miRNA mimics. Compared to negative control, overexpression of miR-125b markedly inhibited 5-FU-induced cytotoxicity in all of the cell lines tested (Fig. 4B). Moreover, overexpression of miR-125b dramatically decreased 5-FU-induced cytotoxicity and increased 5-FU resistance in a miR-125b dose-dependent manner in MCF-7 cells (Fig. 4C). Furthermore, the expression of miR-125b decreased 5-FU-induced cytotoxicity and increased 5-FU resistance under various concentration of 5-FU treatment in MCF-7 cells (Fig. 4D).

**Figure 5:**
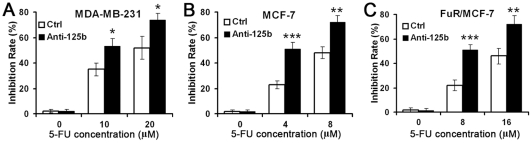
Knockdown of miR-125b sensitized breast cancer cells to chemotherapy. MDA-MB-231 (A), MCF-7 and 5-FU resistant FuR/MCF-7 (B) cells were transfected with 100 nM anti-miR-125b or anti-scramble control (Ctrl). Then the cells were treated with the indicated concentrations of 5-FU for 48 h. Data represented mean ± SD of triplicate experiments. *p<0.05, **p<0.01, ***p<0.001 compared to Ctrl.

Based on the above observation, we further investigated whether reduced miR-125b may sensitize breast cancer cells to chemotherapy. MiR-125b antisense were transfected into MDA-MB-231 (a breast cancer cell line with high endogenous miR-125b), MCF-7 and FuR/MCF-7 (a MCF-7 breast cancer cell line induced to be resistant to 5-FU). Knockdown of miR-125b increased 5-FU induced cytotoxicity in these cells (Fig. 5A and 5B). More importantly, miR-125b antisense decreased IC50 of FuR/MCF-7 to the similar level of wild type MCF-7 (Fig. 5B). These results indicated that miR-125b played an important role in chemotherapeutic resistance in breast cancer cells.

### E2F3 Is a Direct Target of miR-125b in Breast Cancer Cells

**Figure 6:**
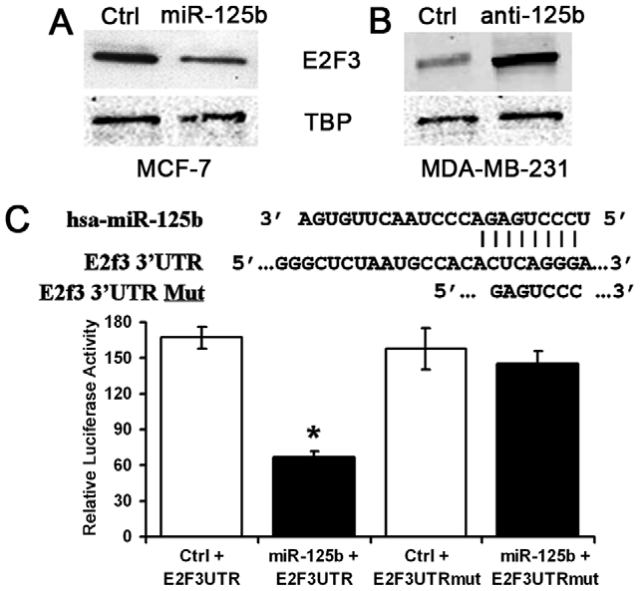
E2F3 was a direct target of miR-125b in breast cancer cells. (A) MCF-7 cells were transfected with 100 nM pre-miR-125b or pre-scramble control (Ctrl). (B) MDA-MB-231 cells were transfected with 100 nM anti-miR-125b or anti-scramble control (Ctrl). Twenty-four hours after transfection, nuclear lysates were prepared for Western blotting with an antibody against E2F3, and TBP was used as a loading control. (C) MCF-7 cells were co-transfected with luciferase reporter plasmids with intact or mutant 3′UTR of E2F3, pre-miR-125 or pre-scramble control (Ctrl). Luciferase activity was measured at 48 h post-transfection using a dual luciferase reporter assay. The results were expressed as relative luciferase activity (firefly/renilla luciferase). Data represented mean ± SD of triplicate experiments. *p = 0.002.

To gain further insight into the function of miR-125b in chemotherapeutic resistance in breast cancer, we used the prediction programs TargetScan Human 5.1 [21], PicTar [22] and DIANA microT v3.0 [23] to identify potential miR-125b target genes. There are 87 predicted targets by all the three prediction algorithms (Table S1). Functional annotation of the 87 highly conserved target genes by DAVID (http://david.abcc.ncifcrf.gov/) revealed enrichment of genes involved with transcription regulation, protein transport, chromatin modification, cell cycle and apoptosis. The three public miRNA databases all predicted that E2F3 might be a target for miR-125b. We were particularly interested in E2F3 because of its essential roles in cell proliferation and apoptosis. Overexpression of miR-125b in MCF-7 cells significantly down-regulated E2F3 protein level (Fig. 6A). In contrast, knockdown of miR-125b up-regulated the expression of E2F3 in MDA-MB-231 cells, which have high expression of endogenous miR-125b (Fig. 6B). These results indicated that E2F3 expression was regulated by miR-125b in breast cancer cells.

To determine if E2F3 is a direct target of miR-125b, a luciferase reporter with E2F 3′ UTR was constructed and co-transfected with miRNA mimics into MCF-7 cells (Fig. 6C). Compared to scramble control, overexpression of miR-125b decreased the luciferase activity of the reporter with E2F3 3′UTR by about 60% (*p* = 0.002, Fig. 6C). Mutations in the seed sequence of miR-125b target site restored the repressed luciferase activity (Fig. 6C), indicating that E2F3 was a direct target of miR-125b.

## Discussion

The expanding knowledge of the molecular pathogenesis of cancer is providing new targets for disease characterization, which might also be used as new markers to select patients for better clinical management. In particular, there are a growing number of studies on miRNAs, which are classified as oncogenes or tumor-suppressor genes and have a pivotal role in progression and prognosis of different tumors [9]. In the present study, we demonstrate that higher circulating miR-125b level is correlated to more advanced ductal carcinoma of the breast and resistance for adjuvant chemotherapy. In addition, miR-125b expression was reversely correlated with apoptosis and proliferation inhibition induced by chemotherapy. Furthermore, breast cancer cells expressing a higher level of miR-125b were more resistant; conversely, reducing miR-125b level sensitized breast cancer cells to chemotherapy. E2F3, as a direct target of miR-125b in breast cancer cells, may be involved in miR-125b-mediated chemotherapeutic response. These data suggest that circulating miR-125b contributes to chemotherapeutic resistance of breast cancer and serves as a potential marker predicting chemotherapeutic response and a target for overcoming chemotherapeutic resistance.

The miRNAs currently analyzed were selected from the comparison of miRNA expression patterns in normal and tumoral breast tissues, as well as from the results of several preclinical studies, suggesting their role in tumor progression and sensitivity [15]–[18], [24], [25]. MiR-125b was reported to have complicated functions as either oncogene-like or tumor suppressor-like in different cancer types or cell lines. Up-regulation of miR-125b is not only observed in pancreatic cancer [26], prostate cancer [27], and acute myeloid leukemia [28], but also promotes cancer cell proliferation and suppresses p53-dependent apoptosis in human neuroblastoma cells [29]. However, miR-125b has been reported to be down-regulated in ovarian carcinoma [30], thyroid carcinoma [31], and oral squamous cell carcinoma [32] and has been shown to inhibit cell proliferation and cell cycle progression in these cancers. Recently, miR-125b was reported to exhibit under-expressed in breast cancer tissues [33], [34]. However, a precise study using microdissection demonstrated that miR-125 was expressed in basal myoepithelial cells but not in luminal ductal cells [35]. The under-expression of miR-125b in breast cancer tissue may be due to loss of myoepithelial cells in cancer tissue instead of downregulation of miR-125b in cancer cells. Moreover, other recent studies reported that miR-125b may act as an oncogene in human breast cancer cells [36] and was related to resistance of breast cancer cells to paclitaxel [37] **.** In our study we show that miR-125b was associated with advanced breast cancer and increased chemotherapeutic resistance, acting primarily as an oncogene. One possible explanation for this controversy is the known diverse nature of miRNA target genes. The net effect of changes in the expression of a miRNA appears to be the sum of all impacts on its targets in a cell type-specific and phenotype-specific manner [38]. The exact association of miR-125b expression and breast tumor progression is still unclear. Further investigations with large patient samples and well designed laboratory studies should be performed in the future to clarify the role of miR-125b in human breast cancer development.

To achieve a more effective and individualised chemotherapeutic treatment of breast cancer patients in the future, it is essential to increase our knowledge in mechanisms responsible for drug resistance, and to define reliable indicators for response to therapy. Commonly accepted prognostic factors are lymph node status, tumor size, histological grade, and patient age. Predictors for the effect of endocrine treatment, currently used in clinical routine, are estrogen and progesterone receptor status, and c-erbB-2 for the effect of trastuzumab [39]. Useful markers for resistance and/or sensitivity of chemotherapy (FEC and/or antracyclin based regimes) have not, so far, been identified. Some markers have shown promising results in a limited number of studies, *e.g.* Ki67 [40], p53 [41], multidrug resistance-associated protein [42] and circulating tumor cells [43], [44]. Recent studies have revealed that a large number of miRNAs are deregulated in drug resistant or sensitive cancer cell lines [45]. For example, Kovalchuk *et al*. [46] found 137 differentially regulated miRNAs (63 upregulated and 75 downregulated) when comparing doxorubicine-resistant and -sensitive breast cancer cell lines. Circulating miRNAs have been identified in the serum/plasma and can be potentially used as biomarkers for tumor characterization and cancer prognosis [11], [47]. To the best of our knowledge, this is the first study investigating the association of circulating miRNAs and chemotherapeutic response. We identified miR-125b as a potential marker predicting chemotherapeutic resistance in breast cancers.

It is well established that miRNAs function via repression of target gene translation. To date, several targets of miR-125b have been identified. One recent study showed that pro-apoptotic Bcl-2 antagonist killer 1 (Bak1) was a direct target of miR-125b, and suppression of Bak1 gene expression through overexpression of miR-125b inhibited Taxol-induced apoptosis and led to an increased resistance to Taxol in breast cancer cells [37]. Tumor suppressor p53 was directly targeted by miR-125b, and overexpression of miR-125b inhibited p53-dependent apoptosis in human neuroblastoma cells and human lung fibroblast cells [48]. A report showed that ErbB2/HER2 and ErbB3/HER3 were both targets of miR-125b in breast cancer SKBr3 cells [49]. However, we could not find a significant association between circulating miR-125 level and HER2 status in breast cancer patients. Consistent with our results, miR-125b did not induce ErbB2/HER2 down-regulation in either prostate cancer cells or breast cancer cells including SKBr3 cells in other reports [27], [37]. Utilizing multiple target prediction tools, we identified 87 potential targets of miR-125b including BMPR1b, an experimentally confirmed target of miR-125b [50] (Table S1). However, several other recently defined targets (MUC1 [51], HER2 [49], BAK1 [37] and ETS1 [17]) are not among our list, reflecting a challenge of miRNA target identification [52]. By combining the bioinformatic and experimental approaches, our results indicated that E2F3 expression was regulated by miR-125b through a target site in the 3′UTR in breast cancer cells. E2F3 family of transcription factors plays a critical role in regulating cell cycle progression and proliferation [53], [54]. Furthermore, E2F3, and in some settings E2F1, induce apoptosis through p53-dependent or -independent pathways [54], [55]. E2F3 pathway has been associated with chemotherapy sensitivity in ER-negative cancers [56]. Thus, it is not surprising that E2F3 be a critical target that mediated the chemotherapeutic resistance in miR-125b overexpressing breast cancer cells.

In conclusion, high miR-125b expression in serum was associated with chemotherapeutic resistance in advanced ductal carcinoma of breast, and the overexpression of miR-125b caused a marked inhibition of anticancer drug activity and increased resistance in breast cancer cells *in vitro*. Because only 5-FU was used in our *in vitro* studies, further investigation will be needed to validate the role of miR-125b with other chemotherapeutic reagents. In addition, we demonstrated that E2F3 was a direct target of miR-125b in breast cancer cells. These data provided evidence that miR-125b may allow stratification for adjuvant therapy, thus offering a potential new biomarker for treatment selection and personalized therapy.

## Materials and Methods

### Ethics Statement

The Study obtained the permission of the ethics committee of Dalian medical university and all patients provided written informed consent.

### Patients

Patients receiving chemotherapy treatment for primary invasive ductal carcinoma of the breast between 01/2009 and 12/2010 at the Department of General Surgery were reviewed. A total of 56 patients (median age 55, range 36–78) were included in the study. All the patients underwent pre-operative neoadjuvant chemotherapy with an association of 5-Florouracil (5-FU), Epirubucin and Cyclophosphamide (FEC) for 4–6 cycles. Before starting the treatment, patients received a baseline blood draw for miRNA expression profiling. Tumor assessment was performed every 3 months by CT-scan and/or chest X-ray coupled with abdomen ultrasound depending on those used at baseline. Subsequently, the patients underwent total mastectomy or breast reservation. The patients with breast reservation received post-operative radiotherapy. Treatment response was assessed by the RECIST criteria [57]. Patients achieving complete (CR) or partial (PR) response were considered as responder; disease stabilization (SD) or disease progression (PD) considered as non-responder. According to the clinical response of neoadjuvant chemotherapy, adjuvant FEC or Taxotere was selectively administered in the patients with PR/CR or PD/SD. Endocrine therapy was administered for patients with positive hormonal receptor. Additionally, 10 age-matched healthy women with no history of cancer and in good health based on self-report were recruited as controls.

### Extraction of total RNA

The *mir*Vana PARIS kit (Ambion) was used to isolate total RNA from human blood serum according to manufacturer's instructions. Four hundred μL of serum samples were incubated with an equal volume of Denaturation Solution for 5 minutes on ice. The RNA extraction was performed by acid-phenol:chloroform, and the precipitation was carried out by ethanol and a filter cartridge. The extracted RNA was eluted in 100 μL of preheated Elution Solution and measured on a NanoDrop ND-1000 Spectrophotometer (Thermo Scientific). The RNA samples were immediately stored at −80°C until use.

### Quantitative real-time PCR

For quantitative real-time PCR, the miRNA-specific TaqMan MicroRNA Assays (Applied Biosystems) for miR16 (reference miR), miR10b, miR34a, miR125b and miR155 were used as described by the manufacturer. Briefly, 100 ng of total RNA was reverse transcribed using primers specific to each miRNA target followed by real-time PCR on a 7900 HT Fast Real-Time PCR System using TaqMan miRNA primers and probes (Applied Biosystems). Triplicate samples, validated endogenous controls and interassay controls were used throughout. MiRNA expression levels were calculated by the ΔCt method: ΔCt  =  mean value Ct (reference miR-16) – mean value Ct (miRNA of interest). The relative expression of miRNA of interest corresponded to the 2^ΔCt^ value.

### Immunohistochemistry and Terminal deoxynucleotidyl transferase (TdT)–mediated dUTP labeling (TUNEL)

The level of PCNA was detected by immunohistochemistry staining in paraffin-embedded tissue sections. Mouse monoclonal anti-PCNA antibody (ab29, Abcam) (1∶6000 dilutions) were used as primary antibody. The mouse IgG was added as the negative control.

TUNEL assay was done using an In situ Apoptosis Detection Kit (R&D Systems). Briefly, after digesting with Protease K, TdT reaction mix was applied to the cells for incubation at 37°C for 60 min, followed by incubation with streptavidin horseradish peroxidase for 10 min. The final reaction of the product was visualized by 3, 39-diaminobenzidine. Approximately 1,000 tumor cells were counted (400×) in each section, and apoptotic index (AI) was expressed as the percentage of TUNEL-positive tumor cells.

The proportion of positive tumor cells was evaluated by two pathologists who were blinded to the study endpoints. For the statistical purpose, we divided cases into two groups: low expression (PCNA≤25% or AI≤1%) and high expression (PCNA>25% or AI>1%) [15].

### Cell culture and drug treatment

Human breast cancer cell lines, MCF-7, T47D, BT20 and MDA-MB-231, were purchased from American Type Culture Collection (ATCC) and grown according to standard protocols. The different inhibitory concentrations of 5-FU were determined by generating dose–response curves after treating the cells with increasing concentrations of 5-FU and analyzing the cell sensitivity using trypan blue dye exclusion assays. Briefly, the cell suspension was appropriately diluted with 0.4% trypan blue dye (Sigma-Aldrich) and hematocytometer was used to estimate the percentage of unstained treated cells compared to the control cells. The inhibition rate was calculated as follows: Inhibition rate = (1-unstained treated cells/control cells) × 100. The IC50 (concentration of 5-FU to produce 50% cell inhibition) was determined and used for further analysis. All experiments were carried in triplicate.

5-FU resistant MCF-7 (FuR/MCF-7) cells were selected in stepwise increasing concentrations beginning at 0.1 µM and ending at 20 µM of 5-FU (Sigma). Cells did not receive next treatment until they had got an 80% confluence after each treatment. Despite massive cell deaths among the sensitive MCF-7 cells under treatment, the cultures were maintained by regular changes of medium intermittently increasing the 5-FU concentration until the surviving cells recovered a normal growth pattern in the medium with 1 µM 5-FU, which was included in the culture medium for MCF-7/5-FU to maintain the drug resistance. The cells were maintained in 5-FU free medium at least 2 weeks before each experiment.

Primary breast cancer cells were isolated from biopsy samples using the Cancer Cell Isolation kit (Panomics) following the manufacturer's protocol. Briefly, tumor pieces of <2 mm size were seeded into six-well culture plates and cultured in RPMI 1640 supplemented with 20% fetal bovine serum, 1% L-glutamine, 1% MEM nonessential amino acid, and 1% antibiotic/penicillin-streptomycin solution. Cells were maintained at 37°C in a humidified atmosphere containing 5% CO2. Cells were harvested with 0.1% trypsin/0.05% EDTA when the cultures had reached ∼80% confluence. These cells were expanded for three to four subsequent passages and thereafter used for determining miR-125b expression level and dose–response curves to 5-FU.

### Luciferase reporter assay and transfection

Full length 3'UTR of E2F3 gene was amplified by PCR and inserted into the firefly luciferase reporter immediately downstream from the stop codon. Mutation in miR-125b target site was introduced by PCR amplification using appropriate oligonucleotides. For luciferase assays, MCF-7 cells were transfected in 6-well plates, using Lipofectamine LTX, with firefly luciferase reporter vectors (0.7 μg), together with a Renilla luciferase control vector (0.07 μg) (Promega). For each well, 25 nM miR-125b mimic or a scramble control miRNA was co-transfected with the reporter constructs. Luciferase activity was measured 24 hrs after transfection using the Dual Luciferase Reporter Assay System (Promega). Firefly luciferase activity was normalized to Renilla luciferase activity.

MiR-125b overexpression and inhibition were achieved by transfection of miRIDIAN miRNA mimic (Thermo Scientific) and LNA antisense oligonucleotides (Exiqon), respectively. Transfection was performed using Lipofectamine RNAiMAX reagent (Invitrogen); reverse transfection procedure was performed according to the manufacture's instructions.

### Western Blot

Nuclear extracts were made using the NE-PER kit (Pierce). E2F3 and TATA binding protein (TBP, nuclear protein loading control) were detected using the anti-E2F3 (N-20, Santa Cruz) and anti-TBP (1TBP18, Abcam) antibodies. For immunoblotting, cell lysates (20 µg) were resolved on 15% SDS–polyacrylamide gels and transferred onto polyvinylidene difluoride membranes (Hybond-P; Amersham Biosciences). Membranes were blocked with 2% non-fat milk for 1 h and then incubated with the primary antibody followed by a horseradish peroxidase-conjugated secondary antibody, and ECL-PLUS Detection System (Amersham Biosciences) was used as substrate for chemiluminescent detection. The band intensity was quantitated using Image J software. Data were normalized to the intensity of TBP bands.

### Statistical analysis

The statistical analyses were performed using the SPSS software package, version 18.0. The chi square or two-tailed Fischeŕs exact test was used to identify potential associations of miRNA concentrations in blood serum with the clinical and histopathological risk factors of the breast cancer patients. For nonparametric comparisons, univariate analyses of the Mann Whitney-U test of two independent variables and bivariate analyses of the Spearman-Rho test were used. All experiments for cell cultures were performed at least triplicate and evaluated by Student's *t* test. Data were presented as mean±standard deviation (SD). *P*<0.05 in all cases was considered statistically significant.

## Supporting Information

Table S1 Predicted targets of miR-125b by all the three prediction algorithms (n = 87)
(PDF)
