# Supplementary material for: Circulating MiR-125b as a Marker Predicting Chemoresistance in Breast Cancer
Source: PLoS One. 2012 Apr 16;7(4):e34210. doi: 10.1371/journal.pone.0034210 (PMC3327688; doi:10.1371/journal.pone.0034210)
Supplement: Table S1 — Predicted targets of miR-125b by all the three prediction algorithms (n = 87) (PDF) [file pone.0034210.s001.pdf]

Table S1. Predicted targets of miR-125b by all the three prediction algorithms (n=87)

Target gene	Gene name	Context score*
Stard13	StAR-related lipid transfer (START) domain containing 13	-1.02
Smek1	SMEK homolog 1, suppressor of mek1 (Dictyostelium)	-0.61
Klhl31	kelch-like 31 (Drosophila)	-0.61
Ier3ip1	immediate early response 3 interacting protein 1	-0.6
Arid3b	AT rich interactive domain 3B (Bright like)	-0.57
Npl	N-acetylneuraminate pyruvate lyase	-0.56
Vps4b	vacuolar protein sorting 4b (yeast)	-0.56
Lactb	lactamase, beta	-0.56
Nup210	nucleoporin 210	-0.55
Atp10d	ATPase, class V, type 10D	-0.55
Lfng	LFNG O-fucosylpeptide 3-beta-N-acetylglucosaminyltransferase	-0.54
Galnt14	UDP-N-acetyl-alpha-D-galactosamine:polypeptide N-acetylgalactosaminyltransferase 14	-0.5
Tmem168	transmembrane protein 168	-0.48
Podxl	podocalyxin-like	-0.47
Tgoln2	trans-golgi network protein 2	-0.45
Cgn	cingulin	-0.43
Mcl1	myeloid cell leukemia sequence 1	-0.42
Foxd2	forkhead box D2	-0.42
Zswim5	zinc finger, SWIM domain containing 5	-0.4
Xkrx	X Kell blood group precursor related X linked	-0.4
Sox11	SRY-box containing gene 11	-0.4
Taf9b	TAF9B RNA polymerase II, TATA box binding protein (TBP)-associated factor	-0.39
Sh3tc2	SH3 domain and tetratricopeptide repeats 2	-0.39
Ube2r2	ubiquitin-conjugating enzyme E2R 2	-0.39
Enpp1	ectonucleotide pyrophosphatase/phosphodiesterase 1	-0.38
Alpk3	alpha-kinase 3	-0.38
Triap1	TP53 regulated inhibitor of apoptosis 1	-0.37
Zfyve1	zinc finger, FYVE domain containing 1	-0.37
Lbh	limb-bud and heart	-0.37
Tbc1d1	TBC1 domain family, member 1	-0.36
Tmem180	transmembrane protein 180	-0.36
Slc35a4	solute carrier family 35, member A4	-0.34
Tsen54	tRNA splicing endonuclease 54 homolog (SEN54, S. cerevisiae)	-0.34
Prdm1	PR domain containing 1, with ZNF domain	-0.34
Cpsf6	cleavage and polyadenylation specific factor 6	-0.34
Msrb3	methionine sulfoxide reductase B3	-0.33
Orc2l	origin recognition complex, subunit 2-like (S. cerevisiae)	-0.33
Ikzf4	IKAROS family zinc finger 4	-0.32
Slc25a15	solute carrier family 25 (mitochondrial carrier ornithine transporter), member 15	-0.32
Kcns3	potassium voltage-gated channel, delayed-rectifier, subfamily S, member 3	-0.32
Abhd6	abhydrolase domain containing 6	-0.31
Phf15	PHD finger protein 15	-0.31
Vtcn1	V-set domain containing T cell activation inhibitor 1	-0.31
Tmem161b	transmembrane protein 161B	-0.31
Rabep2	rabaptin, RAB GTPase binding effector protein 2	-0.31
Map3k11	mitogen-activated protein kinase kinase kinase 11	-0.3
Bmpr1b	bone morphogenetic protein receptor, type 1B	-0.29
Mtf1	metal response element binding transcription factor 1	-0.29

Klc2	kinesin light chain 2	-0.29
Suv39h1	suppressor of variegation 3-9 homolog 1 (Drosophila)	-0.28
Sgpl1	sphingosine phosphate lyase 1	-0.28
Lin28	lin-28 homolog (C. elegans)	-0.27
Mlf2	myeloid leukemia factor 2	-0.27
St8sia4	ST8 alpha-N-acetyl-neuraminide alpha-2,8-sialyltransferase 4	-0.26
Kpna6	karyopherin (importin) alpha 6	-0.26
Ncan	neurocan	-0.26
Tmem26	transmembrane protein 26	-0.26
Rab6b	RAB6B, member RAS oncogene family	-0.25
Kcnip3	Kv channel interacting protein 3, calsenilin	-0.25
Bcl2	B-cell leukemia/lymphoma 2	-0.24
Smurf1	SMAD specific E3 ubiquitin protein ligase 1	-0.24
Suv420h2	suppressor of variegation 4-20 homolog 2 (Drosophila)	-0.23
Nr6a1	nuclear receptor subfamily 6, group A, member 1	-0.23
Snx27	sorting nexin family member 27	-0.23
Sarm1	sterile alpha and HEAT/Armadillo motif containing 1	-0.22
Slc6a17	solute carrier family 6 (neurotransmitter transporter), member 17	-0.22
Cdr2l	cerebellar degeneration-related protein 2-like	-0.2
Kcnc3	potassium voltage gated channel, Shaw-related subfamily, member 3	-0.2
Dtx4	deltex 4 homolog (Drosophila)	-0.19
Dock3	dedicator of cyto-kinesis 3	-0.19
Rab8b	RAB8B, member RAS oncogene family	-0.19
Pafah1b1	platelet-activating factor acetylhydrolase, isoform 1b, beta1 subunit	-0.18
E2f2	E2F transcription factor 2	-0.18
Ppp2r5c	protein phosphatase 2, regulatory subunit B (B56), gamma isoform	-0.17
Fndc3b	fibronectin type III domain containing 3B	-0.17
Ankrd13b	ankyrin repeat domain 13b	-0.16
Eif5a2	eukaryotic translation initiation factor 5A2	-0.15
E2f3	E2F transcription factor 3	-0.13
Mknk2	MAP kinase-interacting serine/threonine kinase 2	-0.12
Brpf1	bromodomain and PHD finger containing, 1	-0.12
Onecut2	one cut domain, family member 2	-0.1
Scn2b	sodium channel, voltage-gated, type II, beta	-0.09
Rc3h2	ring finger and CCCH-type zinc finger domains 2	-0.07
Zbtb7a	zinc finger and BTB domain containing 7a	-0.06
Phox2b	paired-like homeobox 2b	-0.06
Hic2	hypermethylated in cancer 2	-0.02
Setd7	SET domain containing (lysine methyltransferase) 7	0

*Determined by TargetScan Huamn 5.1.
